# Temporal trends and long-term outcomes among recipients of cardiac resynchronization therapy with defibrillator in the United States, 2011–2015: Insights from the National Cardiovascular Data Registry

**DOI:** 10.1016/j.hroo.2022.03.004

**Published:** 2022-04-02

**Authors:** Douglas Darden, Pamela N. Peterson, Xin Xin, Muhammad Bilal Munir, Karl E. Minges, Ilan Goldenberg, Jeanne E. Poole, Gregory K. Feld, Ulrika Birgersdotter-Green, Jeptha P. Curtis, Jonathan C. Hsu

**Affiliations:** ∗ Section of Cardiac Electrophysiology, Division of Cardiology, Department of Medicine, University of California, San Diego, La Jolla, California; † Division of Cardiology, Denver Health Medical Center, Denver, Colorado; ‡ University of Colorado Anschutz Medical Center, Aurora, Colorado; § Center for Outcomes Research and Evaluation, Yale-New Haven Hospital, New Haven, Connecticut; ‖ Section of Cardiovascular Medicine, Yale University School of Medicine, New Haven, Connecticut; ¶ Division of Cardiology, University of Rochester Medical Center, Rochester, New York; # Clinical Cardiovascular Research Center, University of Rochester, Rochester, New York; ∗∗ University of Washington School of Medicine, Seattle, Washington

**Keywords:** Cardiac resynchronization therapy, Implantable cardiac defibrillator, Outcomes research, Registries, Trends

## Abstract

**Background:**

Contemporary data on national trends and outcomes in cardiac resynchronization therapy with defibrillator (CRT-D) recipients following the 2012 updated guidelines has not been studied.

**Objectives:**

This study assessed the trends in long-term outcomes among CRT-D Medicare-aged recipients implanted in 2011–2015.

**Methods:**

Patients aged ≥65 years undergoing de novo CRT-D implantation in the National Cardiovascular Data Implantable Cardiac Defibrillator Registry from 2011–2015 with follow-up through 2017 using Medicare data were included and stratified by year of implant. Patient characteristics, in-hospital outcomes, and outcomes up to 2 years following implant were evaluated.

**Results:**

Among 53,174 patients (aged 75.6–6.4 years, 29.7% women) implanted with CRT-D from 2011 to 2015, there was an increase in implantations based on guideline-concordant recommendations (81.0% to 84.7%, *P* < .001). Compared to 2011, in-hospital procedural complications decreased in 2015 (3.9% vs 2.9%; adjusted odds ratio, 0.76, 95% confidence interval, 0.66–0.88, *P* < .001), driven in part by decreased lead dislodgement (1.4% vs 1.0%). After multivariable adjustment, there was a lower risk of all-cause hospitalization, cardiovascular hospitalization, and mortality at 2-year follow-up in 2015 as compared to 2011, while there were no differences in heart failure hospitalizations at follow-up.

**Conclusion:**

Among Medicare beneficiaries receiving CRT-D from 2011 to 2015, there was an increase in implantations based on guideline-concordant recommendations. Furthermore, there has been a reduction in in-hospital complications and long-term outcomes, including cardiovascular hospitalization, all-cause hospitalization, and mortality; however, there has been no difference in the risk of heart failure hospitalization after adjustment.

**Key Findings**

▪ In this study of 53,174 patients who received a cardiac resynchronization therapy defibrillator (CRT-D) from the National Cardiovascular Data Registry Implantable Cardiac Defibrillator Registry from 2011 to 2015, there was an increase in implantations based on guideline recommendations.
▪ In-hospital procedural complications significantly decreased across the study period.
▪ There were reductions in all-cause hospitalization, cardiovascular hospitalization, and mortality at 2-year follow-up in 2015 as compared to 2011, while there were no reductions in heart failure hospitalization at follow-up.

## Introduction

Cardiac resynchronization therapy with defibrillator (CRT-D) is a standard treatment for select patients with heart failure (HF) and prolonged QRS complex. Multiple trials have shown that by restoring ventricular synchrony, CRT-D improves quality of life, improves functional capacity, and reduces hospitalizations and mortality.^1^, ^2^, ^3^ Since approval by the Food and Drug Administration in 2001, the guidelines have evolved to encompass a broader HF population.^4^ As experience and volume increased early on, several observational studies including patients with CRT-D demonstrated an increase in the rate of implantations, decrease in procedural complications, and improvements in 6-month all-cause and HF hospitalizations, as well as mortality.^5^, ^6^, ^7^, ^8^ While these studies included cohorts up to 2010, there are lacking data on contemporary trends in patient selection and outcomes of CRT-D recipients. Furthermore, the American College of Cardiology/American Heart Association/Heart Rhythm Society (ACC/AHA/HRS) released an update of the 2008 guidelines for device-based therapy for cardiac rhythm abnormalities in 2012 that narrowed class I indications to those with left bundle branch block (LBBB) and QRS ≥150 ms, while expanding the class I indications to also include those with mild HF (New York Heart Association [NYHA] class II).^9^ It remains unknown if the updated guidelines have influenced patient selection and outcomes among CRT-D recipients.

Using data from the Medicare beneficiaries in the National Cardiovascular Data Registry (NCDR) Implantable Cardiac Defibrillator (ICD) Registry between 2011 and 2015, the aims of the study were as follows: (1) to evaluate the trends in the proportions of CRT-D implantations meeting clinical guidelines; (2) to evaluate the trends in in-hospital outcomes; and (3) to evaluate the trends in outcomes up to 2 years.

## Methods

### Data sources

Patients included in this study were enrolled in the NCDR ICD registry, which has been previously described.^10^ The quality of data submitted to the registry is routinely assessed through quality checks, outlier analyses, and site audits. The present study used data collected using the Version 2.1 data collection form. Outcomes following discharge from the index hospitalization were obtained by linking NCDR registry files with corresponding Medicare inpatient fee-for-service claims, as previously described.^11^ Combinations of these identifiers are almost completely unique, enabling identification of registry implantations in the Medicare claims data via International Classification of Diseases, Ninth Revision, and International Statistical Classification of Diseases and Related Health Problems, Tenth Revision, codes, or a combination of data identification methods. Medicare inpatient claims and denominator files were used for follow-up through December 31, 2017, to allow for up to 2 years of follow-up for patients undergoing CRT-D implantation in 2015. The inpatient files contain hospital claims for reimbursement under Medicare Part A. The denominator files contain death dates. Waiver of written informed consent and authorization for NCDR studies were granted by Chesapeake Research Review Incorporated. The research in this study was conducted according to the Helsinki Declaration guidelines on human research.

### Study population

All patients aged 65 years or older who had a registry record for a first-time CRT-D implantation between January 1, 2011, and December 31, 2015, who were enrolled in fee-for-service Medicare at the time of the procedure and could be linked to Medicare claims data were included. Patients with pre-existing devices, those with inconsistent data, and those without linked Medicare data were excluded.

### Outcomes

Implantations based on guideline recommendations for CRT implantation were reported according to the 2012 ACC/AHA/HRS Focused Update on Guidelines for Device-Based Therapy.^9^ “Guideline-concordant” recommendations included all class I indications (NYHA II, III, and ambulatory IV, LVEF ≤35%, QRS ≥150 ms, LBBB) and class II indications (NYHA I, LVEF ≤30%, QRS ≥150 ms, LBBB, ischemic; NYHA II, LVEF ≤35%, QRS 120–149 ms with LBBB or QRS ≥150 ms with non-LBBB; and NYHA III or ambulatory IV with LVEF ≤35%, LBBB with QRS 120–149 ms and non-LBBB with QRS at least 120 ms). “Guideline-discordant” included class III indications, defined as NYHA class I/II status, non-LBBB morphology with QRS duration <150 ms, or any patient with an LVEF ≥35%. For the guideline recommendation analysis only, patients were additionally excluded owing to an independent pacing indication (second- or third-degree heart block or bradycardic cardiac arrest).

In-hospital outcomes included any successful left ventricular (LV) lead placement, intraprocedural death, procedural complication, composite of death and any procedural complication, length of hospitalization >2 days, and optimal medical therapy (OMT) on discharge (defined as beta blocker and angiotensin-converting enzyme (ACE) inhibitor/angiotensin receptor blockade [ARB] prescription). Procedural complications included cardiac arrest, myocardial infarction, cardiac perforation, coronary venous dissection, cardiac tamponade, stroke/transient attack, hematoma, infection requiring antibiotics, hemothorax, pneumothorax, set screw problem, lead dislodgement, conduction block, urgent cardiac surgery, peripheral embolus, and drug reaction. Procedural complications were only available during the index hospitalization.

Long-term outcomes included all-cause mortality, all-cause hospitalization, cardiovascular hospitalization, and HF hospitalization at 30-day, 60-day, 90-day, 1-year, and 2-year time points. Cardiovascular hospitalization was defined as a hospitalization with a primary discharge diagnosis of hypertension, coronary artery disease, myocardial infarction, HF, abdominal or aortic aneurysm, valvular disease, and cardiac arrhythmia.

### Statistical analysis

Recipients of CRT-D were stratified into 5 unmatched cohorts based on the date of implantation (January 1, 2011–December 31, 2011 vs January 1, 2012–December 31, 2012 vs January 1, 2013–December 31, 2013 vs January 1, 2014–December 31, 2014 vs Jan 1, 2015–December 31, 2015). Univariate analysis of continuous variables with normal distributions were compared using 1-way ANOVA testing and continuous variables with nonparametric distributions were compared using the Kruskal-Wallis test. Categorical variables were analyzed with χ^2^ test. For each year of data, the proportion of patients experiencing an event were calculated and examined with the Cochran-Armitage test. To evaluate the independent effect of different time periods, logistic regression and Cox proportional hazards regression models with and without adjustment were used for in-hospital and long-term events, respectively. Odds ratios and hazard ratios (HR) with 95% confidence intervals were reported at years 2012, 2013, 2014, and 2015 vs 2011 (reference).

Variables included in the multivariable analyses adjusted for demographics (age, sex, race), comorbidities (hypertension, cerebrovascular disease, diabetes, chronic lung disease, renal failure–dialysis, atrial fibrillation/flutter, ventricular tachycardia, cardiac arrest, syndromes associated with risk of sudden cardiac death, previous myocardial infarction, previous percutaneous coronary intervention, previous coronary artery bypass grafting, primary valvular heart disease, ischemic heart disease, on maximally tolerated doses of guideline-directed medical therapy), diagnostic information (NYHA class, ejection fraction, QRS duration, systolic blood pressure, body mass index, hemoglobin, creatinine, blood urea nitrogen, sodium, brain natriuretic peptide), discharge medications (aspirin, warfarin, ACE inhibitor, ARB, beta blocker, antiarrhythmic drug), electrophysiology-trained physician, and hospital characteristics (reason for admission [admitted for procedure vs not], teaching status, and geographic location). All potential confounding variables were well represented and collected as part of the NCDR ICD registry. All analyses were performed with SAS version 9.4 (SAS, Cary, NC). The *P* values presented are 2-sided, and *P* < .05 (not adjusted for multiplicity) was considered statistically significant.

## Results

Between January 2011 and December 2015, a total of 53,174 patients aged 65 years and older underwent CRT-D implantation and could be linked to Medicare claims data. Exclusions were based on age <65 years (n = 367,932), non-CRT device or not initial implant (n = 387,600), and unable to link to Medicare claims data (n = 46,130). The cohort was stratified based on year of implant as follows, with no significant increase over the period (*P* trend = .50): 2011, n = 10,833; 2012, n = 10,142; 2013, n = 10,576; 2014, n = 10,601; and 2015, n = 11,022.

### Baseline characteristics

The temporal trends in demographics, comorbidities, diagnostic studies, discharge medications, and hospital-level data are stratified by year and are presented in Table 1. Although differences in several baseline characteristics were statistically significant owing to the large sample size, the absolute differences were modest. The mean age of the entire cohort was 75.6 ± 6.4 years, 29.7% were women, and 87% were white, with no differences across the study period. There was an increase in the rate of nonischemic etiology of HF from 37.1% to 45.7% (*P* < .001), and 91% were primary prevention indication.

**Table 1:** Baseline characteristics of 53,174 Medicare-aged recipients of cardiac resynchronization therapy defibrillator implants, stratified by year of implant

	2011	2012	2013	2014	2015	*P* value
N (% of total)	10,833 (20.37%)	10,142 (19.07%)	10,576 (19.89%)	10,601 (19.94%)	11,022 (20.73%)	
Age at implantation (y), mean (SD)	75.7 (6.3)	75.6 (6.4)	75.5 (6.4)	75.7 (6.4)	75.5 (6.4)	0.5
Age distribution 65–69	2210 (20.4%)	2128 (21.0%)	2206 (20.9%)	2141 (20.2%)	2328 (21.1%)	.03
70–74	2572 (23.7%)	2413 (23.8%)	2621 (24.8%)	2624 (24.8%)	2757 (25.0%)	
75–79	2750 (25.4%)	2555 (25.2%)	2672 (25.3%)	2743 (25.9%)	2832 (25.7%)	
>80	3301 (30.5%)	3046 (30.0%)	3077 (29.1%)	3093 (29.2%)	3105 (28.2%)	
Female	3108 (28.7%)	3014 (29.7%)	3197 (30.2%)	3150 (29.7%)	3354 (30.4%)	.05
Race						
White	9449 (87.2%)	8899 (87.7%)	9189 (86.9%)	9347 (88.2%)	9645 (87.5%)	.01
Hispanic	386 (3.6%)	337 (3.3%)	369 (3.5%)	300 (2.8%)	340 (3.1%)	
Black	793 (7.3%)	697 (6.9%)	804 (7.6%)	740 (7.0%)	795 (7.2%)	
Other	185 (1.7%)	187 (1.8%)	194 (1.8%)	193 (1.8%)	224 (2.0%)	
Reason for admission						
Admitted for this procedure	7478 (69.0%)	7176 (70.8%)	7644 (72.3%)	7845 (74.0%)	8101 (73.5%)	<.001
Cardiac – heart failure	1587 (14.6%)	1331 (13.1%)	1286 (12.2%)	1141 (10.8%)	1290 (11.7%)	
Cardiac – other	1515 (14.0%)	1359 (13.4%)	1398 (13.2%)	1402 (13.2%)	1412 (12.8%)	
Noncardiac	253 (2.3%)	276 (2.7%)	248 (2.3%)	213 (2.0%)	219 (2.0%)	
Prior HF hospitalization						
None	5447 (50.3%)	5221 (51.5%)	5593 (52.9%)	5896 (55.6%)	6242 (56.6%)	<.001
<6 months	2781 (25.7%)	2610 (25.7%)	2682 (25.4%)	2572 (24.3%)	2698 (24.5%)	
≥6 months	2605 (24.0%)	2311 (22.8%)	2301 (21.8%)	2133 (20.1%)	2082 (18.9%)	
NYHA functional class						
I	284 (2.6%)	208 (2.1%)	237 (2.2%)	297 (2.8%)	261 (2.4%)	<.001
II	1562 (14.4%)	1597 (15.7%)	1952 (18.5%)	2160 (20.4%)	2280 (20.7%)	
III	8482 (78.3%)	7923 (78.1%)	7906 (74.8%)	7714 (72.8%)	8038 (72.9%)	
IV	505 (4.7%)	414 (4.1%)	481 (4.5%)	430 (4.1%)	443 (4.0%)	
Etiology and duration						
Ischemic	6817 (62.9%)	6072 (59.9%)	6115 (57.8%)	6047 (57.0%)	5981 (54.3%)	<.001
Nonischemic	332 (3.1%)	291 (2.9%)	347 (3.3%)	341 (3.2%)	402 (3.6%)	
Nonischemic ≥3–9 months	782 (7.2%)	897 (8.8%)	1006 (9.5%)	1156 (10.9%)	1341 (12.2%)	
Nonischemic ≥9 months	2902 (26.8%)	2882 (28.4%)	3108 (29.4%)	3057 (28.8%)	3298 (29.9%)	
Atrial fibrillation/flutter						
None	5960 (55.0%)	5585 (55.1%)	5653 (53.5%)	5610 (52.9%)	5873 (53.3%)	<.001
Paroxysmal	1927 (17.8%)	1813 (17.9%)	2027 (19.2%)	2068 (19.5%)	2124 (19.3%)	
Persistent (>7 days)	687 (6.3%)	563 (5.6%)	772 (7.3%)	789 (7.4%)	917 (8.3%)	
Permanent (>1 year)	1612 (14.9%)	1600 (15.8%)	1582 (15.0%)	1593 (15.0%)	1552 (14.1%)	
Unknown	5960 (55.0%)	5585 (55.1%)	5653 (53.5%)	5610 (52.9%)	5873 (53.3%)	<.001
Ventricular tachycardia						
None	8539 (78.8%)	7995 (78.8%)	8379 (79.2%)	8542 (80.6%)	8846 (80.3%)	<.001
Nonsustained	1546 (14.3%)	1439 (14.2%)	1423 (13.5%)	1301 (12.3%)	1349 (12.2%)	
Sustained	547 (5.0%)	491 (4.8%)	557 (5.3%)	554 (5.2%)	618 (5.6%)	
Unknown	201 (1.9%)	217 (2.1%)	217 (2.1%)	204 (1.9%)	209 (1.9%)	
Cardiac arrest	472 (4.4%)	481 (4.7%)	506 (4.8%)	519 (4.9%)	538 (4.9%)	.33
Syndromes with risk of sudden cardiac death	319 (2.9%)	279 (2.8%)	380 (3.6%)	258 (2.4%)	218 (2.0%)	<.001
Ischemic heart disease	7069 (65.3%)	6365 (62.8%)	6483 (61.3%)	6449 (60.8%)	6534 (59.3%)	<.001
Previous MI and time frame						
No prior MI	5501 (50.8%)	5266 (51.9%)	5592 (52.9%)	5699 (53.8%)	5982 (54.3%)	<.001
MI ≤40 days	283 (2.6%)	217 (2.1%)	219 (2.1%)	205 (1.9%)	236 (2.1%)	
MI >40 days	5049 (46.6%)	4659 (45.9%)	4765 (45.1%)	4697 (44.3%)	4804 (43.6%)	
Previous PCI	3536 (32.6%)	3341 (32.9%)	3494 (33.0%)	3590 (33.9%)	3752 (34.0%)	.12
Previous CABG	4228 (39.0%)	3639 (35.9%)	3765 (35.6%)	3624 (34.2%)	3547 (32.2%)	<.001
Primary valvular heart disease	1921 (17.7%)	1751 (17.3%)	1731 (16.4%)	1718 (16.2%)	1829 (16.6%)	.01
Hypertension	9113 (84.1%)	8586 (84.7%)	9113 (86.2%)	9171 (86.5%)	9544 (86.6%)	<.001
Cerebrovascular disease	1991 (18.4%)	1725 (17.0%)	1968 (18.6%)	1826 (17.2%)	1959 (17.8%)	.01
Diabetes	4568 (42.2%)	4255 (42.0%)	4340 (41.0%)	4468 (42.1%)	4605 (41.8%)	.44
Chronic lung disease	2625 (24.2%)	2431 (24.0%)	2535 (24.0%)	2554 (24.1%)	2544 (23.1%)	.29
End-stage renal disease on dialysis	256 (2.4%)	277 (2.7%)	245 (2.3%)	237 (2.2%)	244 (2.2%)	.10
*Diagnostic studies*						
Ejection fraction						
≤25%	5964 (55.1%)	5585 (55.1%)	5849 (55.3%)	5729 (54.0%)	5979 (54.2%)	.04
26%–34%	3425 (31.6%)	3290 (32.4%)	3341 (31.6%)	3488 (32.9%)	3623 (32.9%)	
35%–54%	1334 (12.3%)	1158 (11.4%)	1279 (12.1%)	1260 (11.9%)	1299 (11.8%)	
≥55%	28 (0.3%)	40 (0.4%)	21 (0.2%)	30 (0.3%)	45 (0.4%)	
Missing	82 (0.8%)	69 (0.7%)	86 (0.8%)	94 (0.9%)	76 (0.7%)	
Ventricular tachycardia ablation	37 (0.3%)	38 (0.4%)	49 (0.5%)	53 (0.5%)	54 (0.5%)	.28
Cardiac rhythm						
Sinus	6624 (61.1%)	6198 (61.1%)	6507 (61.5%)	6511 (61.4%)	6773 (61.4%)	.96
Atrial fibrillation/flutter	2547 (23.5%)	2368 (23.3%)	2538 (24.0%)	2552 (24.1%)	2555 (23.2%)	.45
Atrial tachycardia	42 (0.4%)	44 (0.4%)	61 (0.6%)	59 (0.6%)	58 (0.5%)	.22
Idioventricular	42 (0.4%)	37 (0.4%)	26 (0.2%)	41 (0.4%)	35 (0.3%)	.34
Junctional	77 (0.7%)	89 (0.9%)	84 (0.8%)	114 (1.1%)	70 (0.6%)	<.001
Second-degree block	169 (1.6%)	153 (1.5%)	160 (1.5%)	173 (1.6%)	176 (1.6%)	.94
Third-degree block	365 (3.4%)	346 (3.4%)	464 (4.4%)	616 (5.8%)	725 (6.6%)	<.001
Abnormal intraventricular conduction						
Normal	1614 (14.9%)	1416 (14.0%)	1446 (13.7%)	1324 (12.5%)	1365 (12.4%)	<.001
Left bundle branch block	5641 (52.1%)	5486 (54.1%)	5832 (55.1%)	6035 (56.9%)	6470 (58.7%)	
Right bundle branch block	1290 (11.9%)	1176 (11.6%)	1208 (11.4%)	1233 (11.6%)	1182 (10.7%)	
Delay – nonspecific	2288 (21.1%)	2064 (20.4%)	2090 (19.8%)	2009 (19.0%)	2005 (18.2%)	
QRS duration (ms), mean (SD)	147.6 (26.0)	148.3 (25.6)	149.6 (25.2)	150.4 (25.4)	150.6 (24.9)	<.001
120–129 ms	907 (8.4%)	802 (7.9%)	772 (7.3%)	667 (6.3%)	648 (5.9%)	<.001
130–149 ms	2506 (23.1%)	2299 (22.7%)	2354 (22.3%)	2257 (21.3%)	2370 (21.5%)	
<120 ms	1015 (9.4%)	893 (8.8%)	824 (7.8%)	844 (8.0%)	856 (7.8%)	
≥150 ms	4372 (40.4%)	4264 (42.0%)	4618 (43.7%)	4767 (45.0%)	5163 (46.8%)	
Missing	2033 (18.8%)	1884 (18.6%)	2008 (19.0%)	2066 (19.5%)	1985 (18.0%)	
Systolic blood pressure, mean (SD)	132.8 (22.3)	133.2 (22.7)	132.8 (22.5)	133.0 (22.6)	132.9 (22.5)	.71
Diastolic blood pressure, mean (SD)	72.6 (13.2)	72.9 (13.3)	72.7 (13.1)	72.9 (13.5)	72.9 (13.2)	.33
Body mass index	28.6 (9.8)	28.8 (9.5)	28.8 (9.5)	28.8 (9.1)	28.8 (7.8)	.54
Labs, mean (SD)						
Hemoglobin, g/dL	12.7 (1.9)	12.8 (1.9)	12.8 (1.9)	12.8 (1.8)	12.8 (1.9)	.69
Creatinine, mg/dL	1.4 (1.0)	1.4 (0.9)	1.4 (1.0)	1.4 (0.9)	1.4 (0.9)	.03
Blood urea nitrogen, /dL	27.5 (14.2)	27.1 (14.0)	27.1 (13.9)	27.0 (13.6)	26.8 (13.6)	.01
Sodium, mEq/L	138.5 (5.6)	138.6 (4.6)	138.8 (5.4)	138.6 (5.1)	138.8 (4.9)	<.001
Potassium, mEq/L	4.3 (0.5)	4.3 (0.5)	4.3 (0.5)	4.2 (0.5)	4.3 (0.5)	.20
*Discharge medications*						
ACE inhibitor	6142 (56.7%)	5655 (55.8%)	5712 (54.0%)	5688 (53.7%)	5701 (51.7%)	<.001
Angiotensin receptor blocker	2194 (20.3%)	2233 (22.0%)	2537 (24.0%)	2538 (23.9%)	2773 (25.2%)	<.001
Beta blocker	9588 (88.5%)	9046 (89.2%)	9602 (90.8%)	9566 (90.2%)	10,046 (91.1%)	<.001
Aspirin	7619 (70.3%)	7154 (70.5%)	7386 (69.8%)	7302 (68.9%)	7516 (68.2%)	.005
P2Y12 inhibitor	2585 (23.9%)	2188 (21.6%)	2249 (21.3%)	2138 (20.2%)	2324 (21.1%)	<.001
Amiodarone	1545 (14.3%)	1421 (14.0%)	1598 (15.1%)	1565 (14.8%)	1609 (14.6%)	.19
Sotalol	118 (1.1%)	145 (1.4%)	125 (1.2%)	133 (1.3%)	114 (1.0%)	.07
Dofetilide	48 (0.4%)	52 (0.5%)	69 (0.7%)	50 (0.5%)	71 (0.6%)	.11
Digoxin	2200 (20.3%)	1840 (18.1%)	1735 (16.4%)	1508 (14.2%)	1340 (12.2%)	<.001
Warfarin	3572 (33.0%)	3283 (32.4%)	3537 (33.4%)	3450 (32.5%)	3559 (32.3%)	.35
Diuretic (any)	7782 (71.8%)	7213 (71.1%)	7628 (72.1%)	7616 (71.8%)	7970 (72.3%)	.38
Region						
East North Central	1888 (17.4%)	1709 (16.9%)	1768 (16.7%)	1819 (17.2%)	1753 (15.9%)	<.001
East South Central	821 (7.6%)	755 (7.4%)	792 (7.5%)	784 (7.4%)	810 (7.3%)	
Middle Atlantic	1424 (13.1%)	1266 (12.5%)	1390 (13.1%)	1322 (12.5%)	1321 (12.0%)	
Mountain	358 (3.3%)	409 (4.0%)	430 (4.1%)	466 (4.4%)	561 (5.1%)	
New England	365 (3.4%)	350 (3.5%)	306 (2.9%)	343 (3.2%)	395 (3.6%)	
Pacific	1044 (9.6%)	895 (8.8%)	921 (8.7%)	878 (8.3%)	976 (8.9%)	
South Atlantic	2540 (23.4%)	2460 (24.3%)	2637 (24.9%)	2674 (25.2%)	2687 (24.4%)	
West North Central	1018 (9.4%)	985 (9.7%)	1027 (9.7%)	1049 (9.9%)	1162 (10.5%)	
West South Central	1375 (12.7%)	1313 (12.9%)	1305 (12.3%)	1266 (11.9%)	1357 (12.3%)	
Hospital type						
Government	142 (1.3%)	112 (1.1%)	133 (1.3%)	175 (1.7%)	215 (2.0%)	<.001
Private/community	9629 (88.9%)	9050 (89.2%)	9372 (88.6%)	9285 (87.6%)	9677 (87.8%)	
University	1062 (9.8%)	980 (9.7%)	1071 (10.1%)	1141 (10.8%)	1130 (10.3%)	
Patient beds	462.0 (254.5)	457.2 (259.2)	460.0 (256.7)	457.7 (258.2)	461.7 (256.1)	.53
Teaching	5234 (48.3%)	4805 (47.4%)	4989 (47.2%)	5052 (47.7%)	5137 (46.6%)	.14

ACE = angiotensin-converting enzyme; CABG = coronary artery bypass graft; HF = heart failure; MI = myocardial infarction; NYHA = New York Heart Association; PCI = percutaneous coronary intervention.

Data are n (%) unless indicated otherwise.

Between 2011 and 2015, most comorbidities were stable throughout the period or had small differences. Notably, patients with NYHA class II undergoing CRT-D implantation increased from 14.4% to 20.7%, while those with NYHA III and IV symptoms decreased from 78.3% to 72.9% and 4.7% to 4.0%, respectively (*P* < .001). The proportion of those with LBBB increased from 52.1% to 58.7%, while those with a right bundle branch block decreased slightly from 11.9% to 10.7% (*P* < .001). Patients with a QRS ≥150 ms increased from 40.4% to 46.8% (*P* < .001).

The prescription of ACE inhibitors decreased from 56.7% to 51.7% during the study period, while the prescription of ARBs increased from 20.3% to 25.2% and beta blockers increased from 88.5% to 91.1% (*P* < .001 for all). There was a substantial decrease in the use of digoxin, from 20.3% to 12.2% (*P* < .001).

### Implantations based on guideline recommendations

After an additional exclusion of 3326 patients owing to an independent pacing indication, the cohort for this subanalysis consisted of 49,848 patients. Overall, implants based on guideline-concordant indications increased across the study period (81.0% to 84.7%, *P* < .001), while guideline-discordant indications declined (19.0% to 15.3%, *P* < .001), as seen in Table 2. Furthermore, there was an increase in implants based on class I indications (28.6% to 36.4%, *P* < .001) and decrease in class II recommendations from 52.4% to 48.3% (*P* < .001).

**Table 2:** Cardiac resynchronization therapy defibrillator implantations based on 2012 updated guideline recommendations, stratified by guideline recommendations

	2011	2012	2013	2014	2015	*P* trend
N	10,288	9652	9960	9823	10,125	
Guideline concordant (class I and II)	8331 (81.0%)	7961 (82.5%)	8240 (82.7%)	8280 (84.3%)	8577 (84.7%)	<.001
Class I indications	2944 (28.6%)	2933 (30.4%)	3191 (32.0%)	3345 (34.1%)	3685 (36.4%)	<.001
NYHA II	434 (4.2%)	476 (4.9%)	669 (6.7%)	760 (7.7%)	844 (8.3%)	<.001
NYHA III	2510 (24.4%)	2457 (25.5%)	2522 (25.3%)	2585 (26.3%)	2841 (28.1%)	<.001
Class II indications	5387 (52.4%)	5028 (52.1%)	5049 (50.7%)	4935 (50.2%)	4892 (48.3%)	<.001
Guideline disconcordant (class III)	1957 (19.0%)	1691 (17.5%)	1720 (17.3%)	1543 (15.7%)	1548 (15.3%)	<.001

NYHA = New York Heart Association.

### In-hospital outcomes

The unadjusted crude rates of in-hospital outcomes are presented in Table 3. The proportion of successful LV lead implants remained stable at approximately 96% from 2011 to 2015. Any procedural complication showed a steady decline from 3.9% to 2.9% (*P* < .001). The most common procedural complication was lead dislodgement, which decreased across the study period from 1.4% to 1.0% (*P* = .03). No significant differences in coronary venous dissection, cardiac tamponade, hematoma, or pneumothorax were observed over time. The rates of OMT improved over the study period from 68.1% to 70.6% (*P* < .001) and prolonged hospitalization (greater than 2 days) decreased from 43.8% to 39.2% (*P* < .001). Only 12 procedural deaths were reported during the study period, with no significant change across time.

**Table 3:** Unadjusted crude rates of in-hospital short-term outcomes among cardiac resynchronization therapy defibrillator recipients, stratified by year of implant

	2011	2012	2013	2014	2015	*P* trend
N	10,833	10,142	10,576	10,601	11,022	
*Procedural Outcomes*						
Successful left ventricular lead placement	10,376 (95.8%)	9718 (95.8%)	10,091 (95.4%)	10,099 (95.3%)	10,537 (95.6%)	.04
Reason for failed left ventricular lead implant						
Vascular access	30 (0.3%)	26 (0.3%)	19 (0.2%)	25 (0.2%)	39 (0.4%)	.91
Coronary sinus access	223 (2.1%)	181 (1.8%)	221 (2.1%)	208 (2.0%)	211 (1.9%)	
Tributary vein access	35 (0.3%)	44 (0.4%)	43 (0.4%)	47 (0.4%)	41 (0.4%)	
Coronary sinus dissection	22 (0.2%)	17 (0.2%)	21 (0.2%)	23 (0.2%)	20 (0.2%)	
Unacceptable threshold	23 (0.2%)	19 (0.2%)	19 (0.2%)	16 (0.2%)	18 (0.2%)	
Diaphragmatic stimulation	6 (0.1%)	5 (0.0%)	4 (0.0%)	5 (0.0%)	4 (0.0%)	
Mortality	4 (0.0%)	1 (0.0%)	1 (0.0%)	2 (0.0%)	4 (0.0%)	.09
Any procedural complication	419 (3.9%)	320 (3.2%)	329 (3.1%)	330 (3.1%)	318 (2.9%)	<.001
Cardiac arrest	40 (0.4%)	23 (0.2%)	26 (0.2%)	37 (0.3%)	38 (0.3%)	.21
Myocardial infarction	5 (0.0%)	2 (0.0%)	4 (0.0%)	2 (0.0%)	5 (0.0%)	.69
Cardiac perforation	9 (0.1%)	16 (0.2%)	13 (0.1%)	13 (0.1%)	9 (0.1%)	.43
Coronary venous dissection	34 (0.3%)	23 (0.2%)	25 (0.2%)	32 (0.3%)	27 (0.2%)	.63
Cardiac tamponade	17 (0.2%)	14 (0.1%)	16 (0.2%)	15 (0.1%)	23 (0.2%)	.69
Stroke/transient ischemic attack	6 (0.1%)	5 (0.0%)	5 (0.0%)	10 (0.1%)	8 (0.1%)	.63
Hematoma	51 (0.5%)	29 (0.3%)	46 (0.4%)	35 (0.3%)	42 (0.4%)	.18
Infection requiring antibiotics	12 (0.1%)	6 (0.1%)	4 (0.0%)	1 (0.0%)	5 (0.0%)	.02
Hemothorax	5 (0.0%)	4 (0.0%)	5 (0.0%)	3 (0.0%)	6 (0.1%)	.92
Pneumothorax	45 (0.4%)	41 (0.4%)	51 (0.5%)	49 (0.5%)	38 (0.3%)	.56
Set screw problem	6 (0.1%)	3 (0.0%)	8 (0.1%)	3 (0.0%)	1 (0.0%)	.12
Lead dislodgement	156 (1.4%)	129 (1.3%)	133 (1.3%)	122 (1.2%)	108 (1.0%)	.03
Urgent cardiac surgery	4 (0.0%)	4 (0.0%)	5 (0.0%)	3 (0.0%)	2 (0.0%)	.81
*In-hospital outcomes*						
Length of hospitalization ≥2 days	4742 (43.8%)	4290 (42.3%)	4312 (40.8%)	4182 (39.4%)	4321 (39.2%)	<.001
Optimal medical therapy on discharge†	7380 (68.1%)	7008 (69.1%)	7451 (70.5%)	7504 (70.8%)	7781 (70.6%)	<.001

† Beta blocker and angiotensin-converting enzyme inhibitor or angiotensin receptor blocker.

The unadjusted and multivariable logistic regression analyses for in-hospital outcomes are shown in Table 4. Compared to 2011, no difference in successful LV lead placement was observed over time. There was a decreased risk in any procedural complication (odds ratio [OR] 0.76, 95% confidence interval [CI] 0.66–0.88, *P* < .001) and 8% risk reduction in prolonged hospitalization >2 days (OR 0.92, 95% CI 0.85–0.98, *P* = .02) in 2015 as compared to 2011. Furthermore, compared to 2011, no difference was observed in OMT at discharge after adjustment (OR 1.05, 95% CI 0.99–1.11, *P* = .13).

**Table 4:** Adjusted in-hospital outcomes among cardiac resynchronization therapy defibrillator recipients, stratified by years 2012–2015 compared to 2011 (reference group)

	2011	2012		2013		2014		2015	
		OR (95% CI)	*P* value	OR (95% CI)	*P* value	OR (95% CI)	*P* value	OR (95% CI)	*P* value
Successful LV lead placement	Reference	1.01 (0.88–1.15)	.92	0.91 (0.80–1.04)	.17	0.87 (0.77–1.0)	.05	0.94 (0.82–1.07)	.36
Any procedural complication	Reference	0.81 (0.70–0.93)	<.001	0.79 (0.68–0.91)	<.001	0.80 (0.70–0.93)	<.01	0.76 (0.66–0.88)	<.001
Hospitalization ≥2 days	Reference	0.98 (0.91–1.06)	.64	0.95 (0.89–1.02)	.64	0.95 (0.89–1.02)	.64	0.92 (0.85–0.98)	.02
Optimal medical therapy on discharge†	Reference	1.03 (0.97–1.09)	.39	1.07 (1.01–1.14)	.02	1.07 (1.01–1.14)	.02	1.05 (0.99–1.11)	.13

LV = left ventricular.

† Beta blocker and angiotensin-converting enzyme inhibitor or angiotensin receptor blocker.

### Long-term outcomes

The unadjusted crude temporal trends in long-term outcomes are presented in Table 5. Significant improvements in HF hospitalizations, cardiovascular hospitalizations, and all-cause hospitalizations were observed at 90-day, 1-year, and 2-year follow-up. All-cause mortality only decreased at the 2-year follow-up time point, from 21.7% in 2011 to 16.9% in 2015 (*P* < .001).

**Table 5:** Unadjusted crude rates of long-term outcomes following cardiac resynchronization therapy defibrillator placement, stratified by year of implant

	2011	2012	2013	2014	2015	*P* trend
Mortality						
30 days	153 (1.4%)	129 (1.3%)	107 (1.0%)	127 (1.2%)	143 (1.3%)	.10
90 days	449 (4.1%)	407 (4.0%)	374 (3.5%)	370 (3.5%)	424 (3.8%)	.04
1 year	1342 (12.4%)	1255 (12.4%)	1274 (12.0%)	1252 (11.8%)	1333 (12.1%)	.67
2 years	2356 (21.7%)	2193 (21.6%)	2284 (21.6%)	2197 (20.7%)	1860 (16.9%)	<.001
Hospitalization, all-cause						
30 days	1232 (11.4%)	1122 (11.1%)	1150 (10.9%)	1034 (9.8%)	1058 (9.6%)	<.001
90 days	2448 (22.6%)	2202 (21.7%)	2250 (21.3%)	2065 (19.5%)	2183 (19.8%)	<.001
1 year	4837 (44.7%)	4396 (43.3%)	4521 (42.7%)	4291 (40.5%)	4478 (40.6%)	<.001
2 years	6416 (59.2%)	5809 (57.3%)	6075 (57.4%)	5834 (55.0%)	6110 (55.4%)	<.001
Hospitalization, cardiovascular						
30 days	695 (6.4%)	639 (6.3%)	635 (6.0%)	547 (5.2%)	565 (5.1%)	<.001
90 days	1421 (13.1%)	1265 (12.5%)	1299 (12.3%)	1147 (10.8%)	1189 (10.8%)	<.001
1 year	2846 (26.3%)	2526 (24.9%)	2631 (24.9%)	2409 (22.7%)	2477 (22.5%)	<.001
2 years	3854 (35.6%)	3409 (33.6%)	3601 (34.0%)	3340 (31.5%)	3457 (31.4%)	<.001
Hospitalization, heart failure						
30 days	302 (2.8%)	280 (2.8%)	275 (2.6%)	250 (2.4%)	260 (2.4%)	.12
90 days	703 (6.5%)	636 (6.3%)	644 (6.1%)	579 (5.5%)	634 (5.8%)	.01
1 year	1545 (14.3%)	1407 (13.9%)	1476 (14.0%)	1366 (12.9%)	1450 (13.2%)	.02
2 years	2201 (20.3%)	1979 (19.5%)	2117 (20.0%)	2006 (18.9%)	2082 (18.9%)	.02

**Figure 1:**
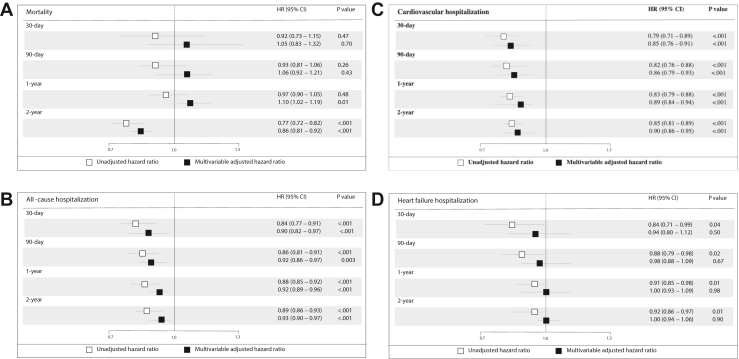
Unadjusted and adjusted long-term outcomes in year 2015 as compared to 2011 (reference) among cardiac resynchronization therapy defibrillator recipients: **A:** mortality; **B:** all-cause hospitalization; **C:** cardiovascular hospitalization; and **D:** heart failure hospitalization.

In multivariable Cox analyses adjusted for potential confounding variables, no difference in the risk of HF hospitalization was observed at 30-day, 90-day, 1-year, or 2-year follow-up in CRT-D implants in year 2015 as compared 2011, while the risk of cardiovascular hospitalization and all-cause hospitalization decreased at each endpoint in long-term follow-up (Figure 1). Compared to 2011, there was a slight increase in 1-year mortality (HR 1.1, 95% CI 1.02–1.19, *P* = .01), while there was an improvement in mortality at 2-year follow-up (HR 0.86, 95% CI 0.81–0.92, *P* < .001).

## Discussion

In this large cohort evaluating the trends in outcomes of Medicare beneficiaries receiving CRT-D from 2011 to 2015, we report several important findings. First, there was an increase in the prevalence of implants based on guideline-concordant indications across the study period. Second, successful LV lead placement was achieved in approximately 96% of patients. Third, in-hospital procedural complications significantly decreased, from 3.9% to 2.9%, driven by a reduction in lead dislodgements. Furthermore, there was a reduction in prolonged hospitalization across the study period. Fourth, there were reductions in the risk of long-term outcomes at 2-year follow-up with cardiovascular hospitalization, all-cause hospitalization, and mortality; however, there was no difference observed in the risk of HF hospitalization.

Clinical trials have influenced guideline recommendations by demonstrating CRT improves morbidity and mortality in select patients with HF largely based on QRS morphology and duration.^1^, ^2^, ^3^ Indeed, careful selection of patients is critical, as some patients, such as those with a right bundle branch block or advanced HF, may not have clinical benefit and implantation carries potential risks. An early report from 2006 to 2008 from the NCDR found nearly 1 in 4 patients receive CRT outside of guideline trial recommendations.^12^ The 2012 guideline update narrowed the class I indications to only include those with an LBBB and QRS duration ≥150 ms, while it also expanded guideline-concordant recommendations to include those with NYHA class II symptoms.^9^ A contemporary update including all patients in the NCDR ICD registry receiving de novo CRT-D implantation from 2012 to 2015 (average age approximately 67 years) showed a significant increase in implants concordant with the updated guidelines, with an increase from 81.2% to 84.2%.^13^ Importantly, the updated guidelines do not include the results of the Biventricular Pacing for Atrioventricular Block and Systolic Dysfunction (BLOCK-HF) trial; therefore, we excluded patients with a pacing indication for this subanalysis.^14^ In our cohort consisting of an older Medicare population, we similarly showed an improvement in the rates of guideline-concordant implants, from 81.0% in 2011 to 84.7% in 2015. Although these are promising data, the elderly are underrepresented in clinical trials and advanced age is a known risk factor for complications associated with CRT.^15^ We show in this analysis that approximately 15% of elderly patients are receiving guideline-discordant CRT implantations, suggesting room for continued improvement in assessing CRT candidacy among older patients.

Complication rates in the clinical trials of CRT ranged from approximately 6% to 13%, driven mostly by lead dislodgements.^2^^,^^3^ In a meta-analysis published in 2011 of 7 CRT randomized trials, lead dislodgement occurred in 5.7% of patients, followed by coronary sinus complications (2%) and pneumothorax (0.9%).^16^ Unlike clinical trials, registries allow large-scale real-world assessment of device complications. Using the Nationwide Inpatient Sample database, Patel and colleagues^6^ noted an overall complication of 16% among CRT-D recipients from 2003 to 2012, with an overall increase across the time period. Individually, mechanical complications (5.9%) were the most common, with an increase from 4.9% to 7.9%, followed by cardiovascular (3.6%), respiratory failure (2.4%), and pneumothorax (1.5%). Since there is no uniform definition of major complications, this overall number may be higher owing to more extensive coding. We expand on prior work with a contemporary and markedly larger cohort with data that are subject to a rigorous event adjudication process with a focus on in-hospital complications. We demonstrate a steady decline from 3.9% to 2.9% (*P* < .001) in any procedural complication from 2011 to 2015. The most common procedural complication was lead dislodgement, which decreased across the study period from 1.4% to 1.0% (*P* = .03). It remains uncertain if the improvement is based on greater implanter experience and skill or advancements in delivery systems and leads.^17^^,^^18^ Nonetheless, these generalizable contemporary complication rates can inform the shared decision-making process in considering CRT implantation among the elderly.

We observed temporal trend improvements in several long-term outcomes at 2 years, including all-cause hospitalization, cardiovascular hospitalization, and mortality; however, no improvement was observed in HF hospitalizations at any point in follow-up. Prior reports from the NCDR ICD registry demonstrated a reduction in all-cause rehospitalization among Medicare beneficiaries who underwent ICD implantation (including CRT-D) between 2006 and 2010, driven mainly by HF rehospitalization at 6 months (13.1% to 11.4%).^8^ Conversely, contemporary reports including cohorts up to 2018 from the National Inpatient Sample and the National Readmission Database have shown an increase in HF hospitalizations and readmissions in recent years, respectively.^19^^,^^20^ While CRT has been shown to reduce HF hospitalizations by 37% in select patients, it would be suspected that risk of HF hospitalizations would decrease with parallel improvements in guideline indication adherence.^21^ Although our study was not equipped to determine the underlying reasons behind failing to reduce risk of HF hospitalizations, several plausible explanations are worth expanding on. Roughly 70% of patients were prescribed OMT (beta blocker and ACE inhibitor or ARB) on discharge with no improvement across the study period. Early reports from the NCDR ICD registry demonstrated an increase of OMT prescription in 2010 compared to 2006; however, nearly a quarter of patients still did not receive OMT, which was still higher than our contemporary and older cohort.^7^ While the low rate of ACE/ARB prescription in our cohort may be partially explained by increased use of angiotensin-neprilysin inhibitors, as it was not captured in the registry, a prior study showed only 2% of eligible patients were prescribed angiotensin-neprilysin inhibitors in a large national registry following approval in 2015.^22^^,^^23^ Moreover, the Centers for Medicare and Medicaid Services implemented the Federal Hospital Readmissions Reduction Program in 2012 to provide financial incentive to hospitals to reduce readmissions, which has been shown to decrease 30-day and 1-year HF hospitalizations.^24^^,^^25^ While it may not be expected to achieve a beneficial effect of CRT leading to reduction in HF hospitalization in the immediate follow-up periods, still no difference was observed after both implementation of the Hospital Readmissions Reduction Program and 2012 updated guidelines, with 1 in 5 patients experiencing a HF hospitalization at the extended follow-up period of 2 years. Lastly, there may have been variability in coding or even miscoding of HF hospitalization in our patient cohort. Still, this may call for outpatient strategies to reduce HF hospitalization risk, such as multidisciplinary HF clinics, remote monitoring adherence, or use of virtual follow-up specifically for the aging population.

Lastly, a significant improvement in mortality was observed after adjustment only at 2-year follow-up, with a decrease from 21.7% in 2011 to 16.9% in 2015. The risk of death in our cohort is much higher than reported in clinical trials, as would be expected given the advanced age in our cohort. For instance, the MADIT-CRT trial reported 6.8% mortality rate in the CRT-D group at an average follow-up of 2.4 years.^3^ In a separate study using the NCDR ICD registry, Borne and colleagues^8^ reported a reduction in 6-month mortality among CRT-D Medicare beneficiary recipients from 8.0% in 2006 to 6.6% in 2010. Reasons for lack of reduction in mortality risk at earlier endpoints in our contemporary cohort are unclear. It may be that CRT-D itself is not the primary driver in mortality reduction given that there is no difference in HF hospitalizations. Rather, improvements in areas such as management of comorbidities, patient selection resulting in less-frail patients, and overall closer outpatient follow-up may play an important role in the aging population with CRT-D.

### Study limitations

Our study must be interpreted in the context of several limitations. The study was observational by design and causation should not be assumed. Outcomes can only be adjusted for variables captured in the registry, and limited information exists on the clinical decision-making for accepting or rejecting a particular therapy. This study should be interpreted as hypothesis-generating and provides valuable insight into the real-world practice-changing patterns. Second, the study was limited to Medicare beneficiaries. The generalizability of our results to beyond the Medicare population remains unclear. Lastly, we were unable to assess long-term outcomes related to optimal medical therapy in the follow-up period, device programming information, or device complications owing to unavailability and inconsistent coding related to Medicare codes spanning our study period.

## Conclusion

In a large, contemporary registry of Medicare patients receiving CRT-D from 2011 to 2015, we observed improved adherence to guideline-concordant device implantation based on updated guideline-recommended indications, decrease in in-hospital complications, and improvements in all-cause hospitalization, cardiovascular hospitalization, and mortality at 2-year follow-up. However, there was no improvement in the risk of HF hospitalization.

## References

[1] Bristow M.R., Saxon L.A., Boehmer J. (2004). Cardiac-resynchronization therapy with or without an implantable defibrillator in advanced chronic heart failure. N Engl J Med.

[2] Tang A.S., Wells G.A., Talajic M. (2010). Cardiac-resynchronization therapy for mild-to-moderate heart failure. N Engl J Med.

[3] Moss A.J., Hall W.J., Cannom D.S. (2009). Cardiac-resynchronization therapy for the prevention of heart-failure events. N Engl J Med.

[4] Moynahan M., Faris O.P., Lewis B.M. (2005). Cardiac resynchronization devices: the Food and Drug Administration's regulatory considerations. J Am Coll Cardiol.

[5] Sridhar A.R., Yarlagadda V., Parasa S. (2016). Cardiac resynchronization therapy: US trends and disparities in utilization and outcomes. Circ Arrhythm Electrophysiol.

[6] Patel N., Viles-Gonzalez J., Agnihotri K. (2018). Frequency of in-hospital adverse outcomes and cost utilization associated with cardiac resynchronization therapy defibrillator implantation in the United States. J Cardiovasc Electrophysiol.

[7] Dodson J.A., Lampert R., Wang Y., Hammill S.C., Varosy P., Curtis J.P. (2014). Temporal trends in quality of care among recipients of implantable cardioverter-defibrillators: insights from the National Cardiovascular Data Registry. Circulation.

[8] Borne R.T., Peterson P.N., Greenlee R. (2014). Temporal trends in patient characteristics and outcomes among Medicare beneficiaries undergoing primary prevention implantable cardioverter-defibrillator placement in the United States, 2006-2010. Results from the National Cardiovascular Data Registry's Implantable Cardioverter-Defibrillator Registry. Circulation.

[9] Epstein A.E., DiMarco J.P., Ellenbogen K.A. (2013). 2012 ACCF/AHA/HRS focused update incorporated into the ACCF/AHA/HRS 2008 guidelines for device-based therapy of cardiac rhythm abnormalities: a report of the American College of Cardiology Foundation/American Heart Association Task Force on Practice Guidelines and the Heart Rhythm Society. J Am Coll Cardiol.

[10] Messenger J.C., Ho K.K., Young C.H. (2012). The National Cardiovascular Data Registry (NCDR) data quality brief: the NCDR Data Quality Program in 2012. J Am Coll Cardiol.

[11] Hammill B.G., Hernandez A.F., Peterson E.D., Fonarow G.C., Schulman K.A., Curtis L.H. (2009). Linking inpatient clinical registry data to Medicare claims data using indirect identifiers. Am Heart J.

[12] Fein A.S., Wang Y., Curtis J.P. (2010). Prevalence and predictors of off-label use of cardiac resynchronization therapy in patients enrolled in the National Cardiovascular Data Registry Implantable Cardiac-Defibrillator Registry. J Am Coll Cardiol.

[13] Sandhu A., Bao H., Minges K.E. (2019). Use of cardiac resynchronization therapy defibrillator in US hospitals. JAMA Cardiol.

[14] Curtis A.B. (2013). Biventricular pacing for atrioventricular block and systolic dysfunction. N Engl J Med.

[15] Bilchick K.C., Kamath S., DiMarco J.P., Stukenborg G.J. (2010). Bundle-branch block morphology and other predictors of outcome after cardiac resynchronization therapy in Medicare patients. Circulation.

[16] van Rees J.B., de Bie M.K., Thijssen J., Borleffs C.J., Schalij M.J., van Erven L. (2011). Implantation-related complications of implantable cardioverter-defibrillators and cardiac resynchronization therapy devices: a systematic review of randomized clinical trials. J Am Coll Cardiol.

[17] Cheng A., Wang Y., Curtis J.P., Varosy P.D. (2010). Acute lead dislodgements and in-hospital mortality in patients enrolled in the National Cardiovascular Data Registry implantable cardioverter defibrillator registry. J Am Coll Cardiol.

[18] Erath J.W., Benz A.P., Hohnloser S.H., Vamos M. (2019). Clinical outcomes after implantation of quadripolar compared to bipolar left ventricular leads in patients undergoing cardiac resynchronization therapy: a systematic review and meta-analysis. Europace.

[19] Khan M.S., Sreenivasan J., Lateef N. (2021). Trends in 30- and 90-day readmission rates for heart failure. Circ Heart Fail.

[20] Salah H.M., Khan Minhas A.M., Khan M.S. (2022). Trends in hospitalizations for heart failure, acute myocardial infarction, and stroke in the United States from 2004-2018. Am Heart J.

[21] McAlister F.A., Ezekowitz J., Hooton N. (2007). Cardiac resynchronization therapy for patients with left ventricular systolic dysfunction: a systematic review. JAMA.

[22] McMurray J.J., Packer M., Desai A.S. (2014). Angiotensin-neprilysin inhibition versus enalapril in heart failure. N Engl J Med.

[23] Luo N., Fonarow G.C., Lippmann S.J. (2017). Early adoption of sacubitril/valsartan for patients with heart failure with reduced ejection fraction: insights from Get With the Guidelines-Heart Failure (GWTG-HF). JACC Heart Fail.

[24] McIlvennan C.K., Eapen Z.J., Allen L.A. (2015). Hospital readmissions reduction program. Circulation.

[25] Gupta A., Allen L.A., Bhatt D.L. (2018). Association of the hospital readmissions reduction program implementation with readmission and mortality outcomes in heart failure. JAMA Cardiol.

